# East China Sea Storm Surge Modeling and Visualization System: The Typhoon Soulik Case

**DOI:** 10.1155/2014/626421

**Published:** 2014-10-14

**Authors:** Zengan Deng, Feng Zhang, Linchong Kang, Xiaoyi Jiang, Jiye Jin, Wei Wang

**Affiliations:** ^1^Key Laboratory of Digital Ocean, State Oceanic Administration, Tianjin 300171, China; ^2^National Marine Data and Information Service, Tianjin 300171, China

## Abstract

East China Sea (ECS) Storm Surge Modeling System (ESSMS) is developed based on Regional Ocean Modeling System (ROMS). Case simulation is performed on the Typhoon Soulik, which landed on the coastal region of Fujian Province, China, at 6 pm of July 13, 2013. Modeling results show that the maximum tide level happened at 6 pm, which was also the landing time of Soulik. This accordance may lead to significant storm surge and water level rise in the coastal region. The water level variation induced by high winds of Soulik ranges from −0.1 to 0.15 m. Water level generally increases near the landing place, in particular on the left hand side of the typhoon track. It is calculated that 0.15 m water level rise in this region can cause a submerge increase of ~0.2 km^2^, which could be catastrophic to the coastal environment and the living. Additionally, a Globe Visualization System (GVS) is realized on the basis of World Wind to better provide users with the typhoon/storm surge information. The main functions of GVS include data indexing, browsing, analyzing, and visualization. GVS is capable of facilitating the precaution and mitigation of typhoon/storm surge in ESC in combination with ESSMS.

## 1. Introduction

**Figure 1:**
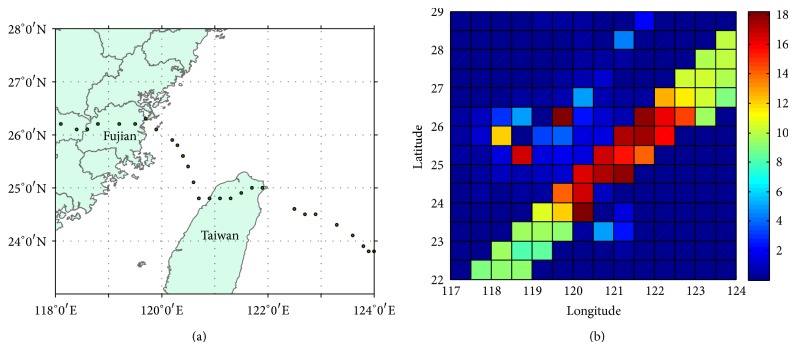
(a) Trajectory of Typhoon Soulik. The cycle refers to the core of Typhoon; (b) a snapshot of HY-2 satellite observed wind speed at the time of Typhoon Soulik landing the Fujian coastal region (units: m/s).

East China Sea (ECS), in particular the area north of Taiwan Island, is greatly vulnerable to hurricane/typhoon events. Several major hurricanes and the associated storm surges attack this region for every single year, which result in huge economic loss. The storm surge-induced inundation has great impacts/threats on the activities and even the safety of the people living in the coastal region. For example, totally 24 storm surge events hit the coastal regions of south and East China Sea in 2012, caused economic losses of more than ¥12.6 billion, and caused the death of 9 people (disaster reports of State Oceanic Administration of China, http://www.soa.gov.cn/zwgk/hygb/zghyzhgb/zhgb/201303/t20130306_24224.html). Typhoon Soulik was developed in ECS in July 13, 2013, with maximum wind speed of over 30 m/s. Soulik hit north Taiwan Island (121.1°E, 24.8°N) at 7 am in July 13 and eventually landed on the coastal region (119.2°E, 26.2°N) of Fujian Province, China, at 6 pm that evening (Figure 1(a) shows Soulik's moving trajectory). Preliminary estimates in the Fujian Province indicated that 72 million people were affected by the storm. At least 990 homes collapsed and direct economic losses reached ¥1.744 billion (US$284.2 million). Given that, storm surge prediction and rapid disaster information service are of vital importance to evacuation and mitigation.

In this study the ECS Storm Surge Modeling System (ESSMS) has configured using Regional Ocean Modeling System (ROMS [1]) for modeling the regional storm surge events. Case study of Typhoon Soulik-induced storm surge was conducted by utilizing ESSMS. The effect of storm surge on the water level over ECS was investigated through sensitivity experiments. To better provide users with the typhoon/storm surge information, a Globe Visualization System (GVS) is also developed based on World Wind, an open source software proposed by NASA. GVS is mainly used for indexing, browsing, analyzing, and displaying the disaster information. Combined with ESSMS, GVS can better facilitate the precaution and mitigation of typhoon/storm surge in ESC in the future by providing the disaster information.

## 2. Formulations of ESSMS

Brief description of ESSMS formulation in this section is given by following ROMS technical manual [2]. ROMS is a three-dimensional, free-surface, terrain-following numerical model with hydrostatic and Boussinesq assumptions. The governing equations in Cartesian coordinates are

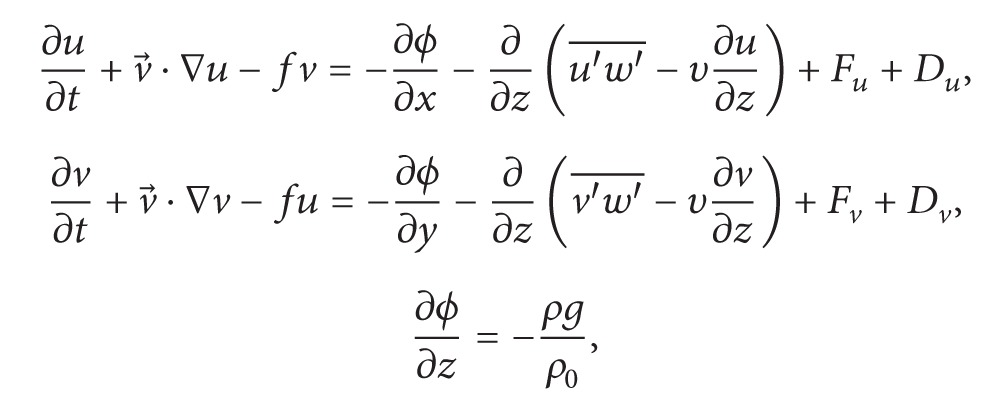
(1)

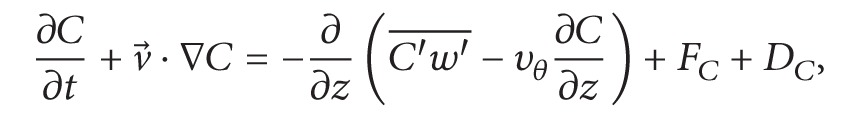
(2)

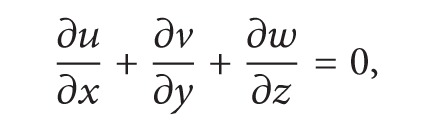
(3)

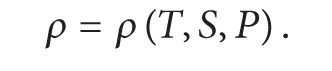
(4)


Equation (1) is momentum equation, (2) accounts for the time evolution of the scalar concentration field, (3) is the continuity equation, and (4) is the equation of state. In those equations, *u*, *v*, and *w* are the (*x*, *y*, *z*) components of velocity vector $\vec{v}$; *C* is scalar quantity such as temperature and salinity; *ρ* is water density; *ϕ* is dynamic pressure; *D* is the optional horizontal diffusive term; *F* is the source term which includes the tidal forcing; $\overset{-}{u^{\prime }w^{\prime }}$ and $\overset{-}{v^{\prime }w^{\prime }}$ are Reynolds stresses which adopt the wind forcing as the top boundary condition and the bottom fraction as the bottom boundary condition. For more descriptions of the equations please refer to ROMS technical manual.

## 3. Configurations of ESSMS

ESSMS is compiled using Intel compiler on a Linux (Red Hat 6) environment. The modeling domain for Soulik case is from 118°E to 122°E in longitude and from 24°N to 27°N in latitude, with horizontal resolution of 1/64°. 22 terrain-following layers are specified in the vertical direction. ETOPO2 data is introduced to represent the topography. WOA05 (World Ocean Atlas 2005) climatology is used to initialize the model. Atmospheric forcings, that is, air temperature, radiation flux, short wave radiation, precipitation, and water vapor mixing ratio, are from COADS (Comprehensive Ocean-Atmosphere Data Set). Multi-year-mean 6-hourly CCMP (Cross-Calibrated Multiplatform [3]) satellite-based analysis winds are adopted to force the spin-up run (from January 1, 2013, to July 12, 2013). The storm surge run (from 1 to 24 o'clock in July 13, 2013) is forced by HY-2 satellite observational winds provided by National Satellite Ocean Application Service of China. 10 tidal forcing components (M_2_, S_2_, N_2_,  K_2_, K_1_, O_1_, P_1_, Q_1_, M_f_  and M_m_) are specified at the open boundary. The tidal forcing is derived from the tidal dataset TPXO7 (http://ceoas.oregonstate.edu/research/).

## 4. Experiments and Modeling Results

After the spin-up run (from January 1, 2013, to July 12, 2013), two experiments have been conducted in July 13, 2013, when Typhoon Soulik happened. Experiment  1 still uses the averaged CCMP winds, serving as the coordinate run whereas Experiment  2 adopts HY-2 satellite observational winds to simulate the Typhoon Soulik-induced storm surge.

**Figure 2:**
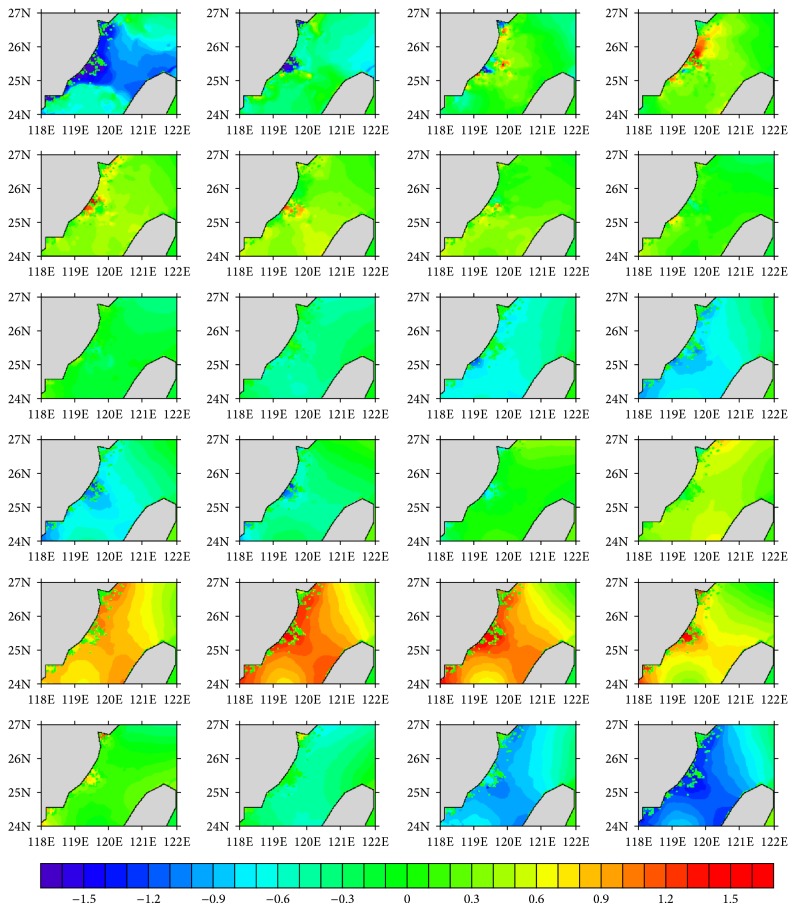
Sea level series in July 13, 2013; output from Experiment  1 which is forced by CCMP multiyear mean winds. (Units: m; from left to right and from top to bottom, the plot, resp., presents the situation of 1, 2, 3, …, 24 o'clock.)

**Figure 3:**
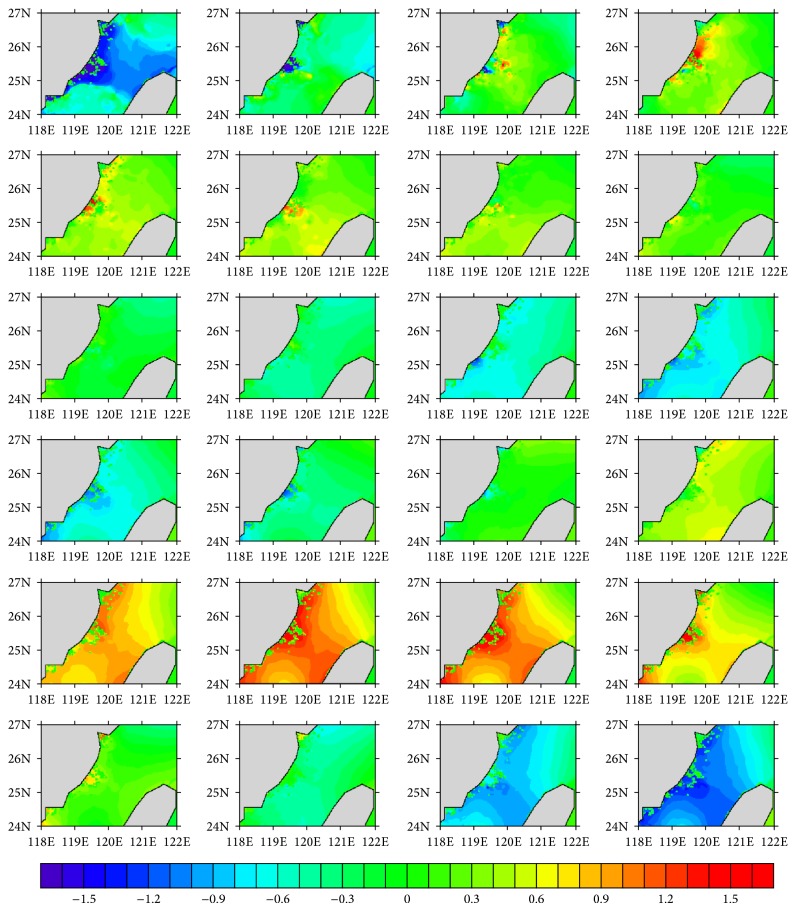
Sea level series in July 13, 2013. Output from Experiment  2 which is forced by HY-2 winds. (Units: m; from left to right and from top to bottom, the plot, resp., presents the situation of 1, 2, 3, …, 24 o'clock.)

**Figure 4:**
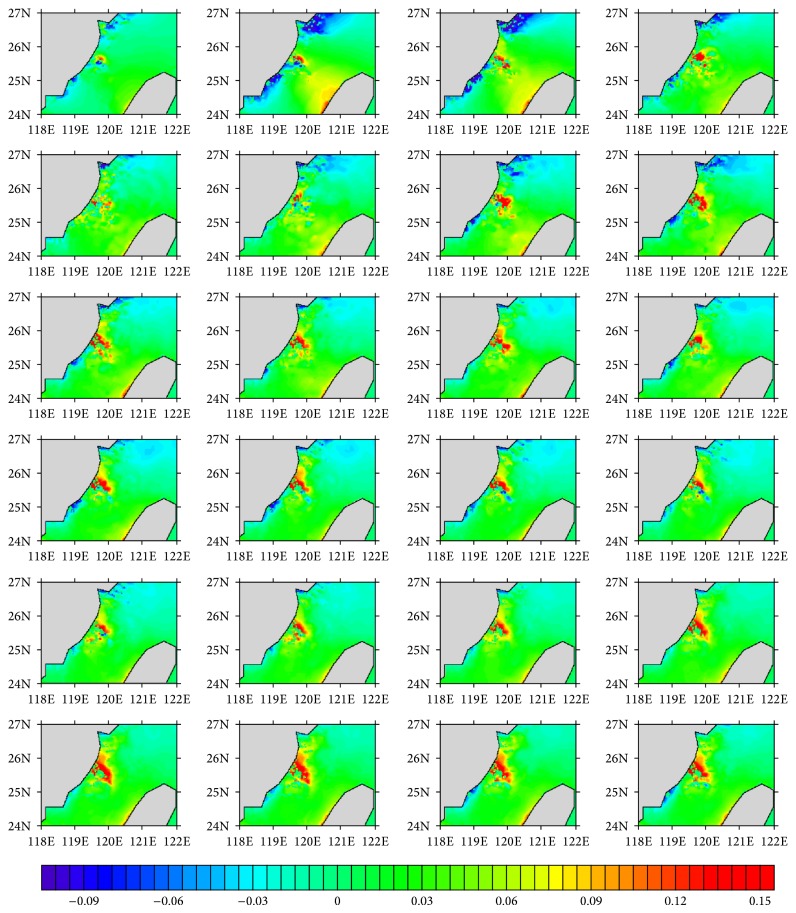
Sea level differences between Experiments  1 and 2. (Units: m; from left to right and from top to bottom, the plot, resp., presents the situation of 1, 2, 3, …, 24 o'clock.)

The hourly water level series for Experiments  1 and  2 are, respectively, shown in Figures 2 and 3, and the Soulik resulted water level change is given in Figure 4. Daily cycle of the tide-resulted water level fluctuation is presented by both of the experiments. The water level generally varies in the range of −1.5~1.5 m, with two high tides in a day, which agrees with the characteristic of semidiurnal tide. The maximum tide level happened at 6 pm, which was also the landing time of Soulik. This accordance may result in significant storm surge in the coastal region. The water level variation induced by high winds of Soulik varies from −0.1 to 0.15 m, with the maximum water level increase appearing on the left hand side of typhoon track, which is caused by the wind setup, near the landing place (Figure 4), causing inundation in the coasts. It is calculated that 0.15 water level rise can lead to ~0.2 km^2^ submerge increase in this region, which would cause vital impacts on coastal environment and people living.

## 5. GVS and Visualization of Soulik

### 5.1. Description of GVS

**Figure 5:**
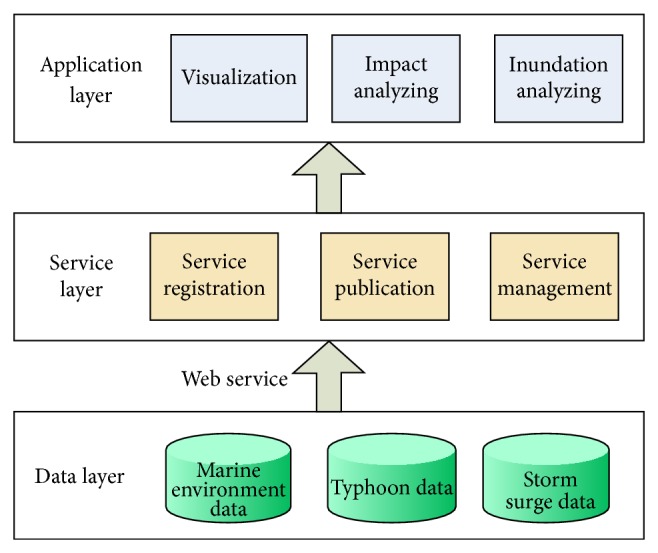
Framework of GVS server.

GVS is designed with a client/server manner using Java language. The GVS's server consists of three portions: data layer, service layer, and application layer (Figure 5). The data layer includes databases of marine environment, typhoon, and storm surge. Marine environment database stores background environmental information such as wind, current, wave, temperature, salinity, bathymetry, and coastline. Typhoon database contains track, radius, wind speed, wind direction, air pressure, and cloud coverage. All the ESSMS simulated storm surge variables are stored in the storm surge database, including water level, current, temperature, and salinity within the modeling region. In the storm surge modeling process, ESSMS reads the winds from typhoon database and geographic/oceanographic data from marine environment database and then sends the simulated storm surge data to storm surge database. Service layer serves as a basic interface/service platform, which realizes the link and communications between data layer and application layer through registration and publication of the request services from application layer. There are three functional modules in application layer, visualization module, typhoon impacts analyzing module, and inundation analyzing module, respectively, used for displaying environmental data on the globe, analyzing the impacts along the typhoon track, and analyzing the inundation area/impacts induced by storm surge.

**Figure 6:**
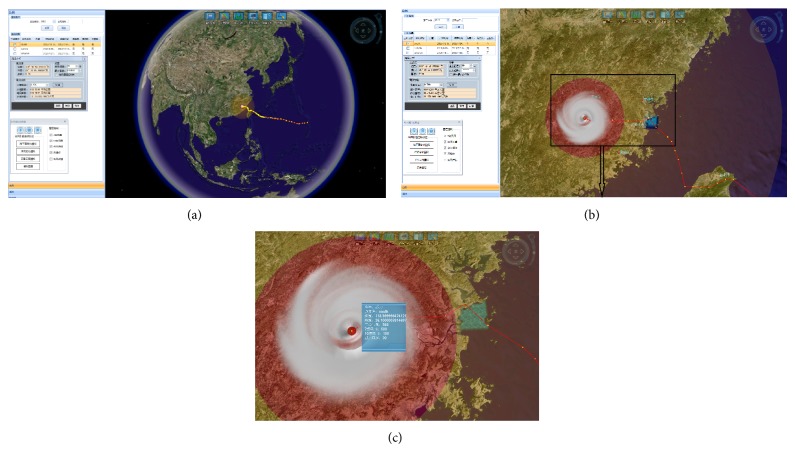
Layout of GVS client and typhoon visualization.

Another outstanding characteristic of GVS is the globe-based visualization, which was adopted in an oil spill information service system [4]. The layout of the GVS's client is shown in Figure 6. The globe is used for three-dimensional visualization and the menu bar provides functions of data/information searching, indexing, browsing, and analyzing. All kinds of marine data that have geographic information/spatial coordinates can be loaded into this system, as well as displayed, in figure or animation, on the globe. In particular, typhoon track, radius, wind vector, and water level and inundation area can all be well displayed on the globe.

### 5.2. Visualization of Soulik

**Figure 7:**
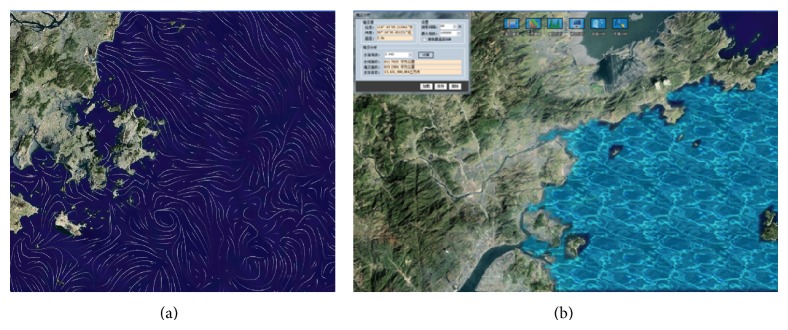
Current visualization (a) and water level/inundation visualization (b).

Using the simulations of ESSMS here we present the functions of GVS with respect to visualization and analysis. The background marine environmental variables, such as sea level, currents, winds, and water temperature, in the typhoon/storm surge region can be visualized on the globe using contour or vector (Figure 7(a)). Then the typhoon information is overlapped, including track (geographic coordinate), time, radius, and wind speed of the core. This information can be both displayed on the globe and listed in a table (Figure 6(c)). A typhoon impact analyzing module is provided accompanying typhoon visualization for estimating the impact area/degree. With this module, the impact area as well as the environment information along the typhoon track can be obtained.

The visualizing and analyzing of storm surge-induced inundation is illustrated in Figure 7(b). In the inundation visualization, water of storm surge appeared as blue with light effect. The submerge area is presented on the globe. In addition, an inundation analyzing module (see the menu bar on the top-left of Figure 7(b)) is designed to calculate the submerge area using the modeled water level. The marine environmental information and constructions within the submerge area can also be provided.

## 6. Conclusions

We first configured ESSMS using a sophisticated ocean model ROMS in ECS to simulate the typhoon-induced storm surge. Case simulation of Typhoon Soulik demonstrates that the modeled storm surge is generally realistic. The visualization of typhoon and inundation is our main focus in this study. Therefore GVS is then developed based on open source software World Wind. With the example storm surge data simulated by ESSMS, the visualization and analysis functions/characteristics of GVS are presented. It is illustrated that GVS is capable of providing timely information of typhoon track, radius, wind speed, and storm surge water level and inundation area. Additionally, all the marine environmental data and constructions within the typhoon/storm surge affected region can be easily obtained through GVS. It is believed that GVS is of practical help to disaster precaution and mitigation.
